# Sports-related concussion: assessing the comprehension, collaboration, and contribution of chiropractors

**DOI:** 10.1186/s12998-022-00471-z

**Published:** 2022-12-27

**Authors:** Nicholas Shannon, Jon Patricios

**Affiliations:** 1 Faculty of Health Sciences, School of Clinical Medicine, University of the Witwatersrand, Johannesburg, South Africa; 2 Melbourne Chiropractic and Sports Care, Shannon Clinic, Melbourne, Australia

**Keywords:** Sport, Concussion, Chiropractic, Cervical spine, Head impact

## Abstract

Over the last 2 decades, sports-related concussion (SRC) awareness and management have evolved from an emphasis on complete cognitive and physical rest to evidence-based protocols and interventions. Chiropractors are primary care providers with exposure to athletes and teams in collision sports and, in addition, manage patients with concussion-like symptoms including neck pain, dizziness, and headache. With SRC frequently occurring in the absence of a medical practitioner, the role of allied health practitioners like chiropractors should be emphasised when it comes to the recognition, assessment, and management of SRC. This commentary discusses the potential contribution of chiropractors in SRC and the specific role their expertise in the cervical spine may play in symptom evaluation and management. A PubMed and Google scholar review of the chiropractic SRC literature suggests that the chiropractic profession appears under-represented in concussion research in athletic populations compared to other medical and allied health fields. This includes an absence of chiropractic clinicians with a focus on SRC participating in the Concussion in Sport Group (CISG) and the International Consensus Conferences on Concussion. Furthermore, with evolving evidence suggesting the importance of cervicogenic manifestations in SRC, there is an opportunity for chiropractors to participate in SRC diagnosis and management more fully and contribute scientifically to an area of specialised knowledge and training. With a dearth of chiropractic orientated SRC science, clinical SRC expertise, and clinical chiropractic representation in the CISG; it is incumbent on chiropractic clinicians and scientists to take up this opportunity through meaningful contribution and involvement in the SRC field.

## Background

Our awareness of sport related concussion (SRC) has evolved significantly in the last 2 decades due to an exponential increase in research and publications, greater interdisciplinary collaboration driven by the Concussion in Sport Group (CISG) and resultant evidence-based protocols and interventions [1–3]. Apart from clinical training in SRC recognition and management, there are now education programs to improve coaches’, parents’, and athletes’ awareness of concussion symptoms; strict “no return to play on the same day” rules, screening assessment tools, and guidelines for return to play and learn [3, 4]. Moreover, the historical approach of “resting in a dark room” has been replaced by domain-based targeted management approaches to both alleviate symptoms and reduce recurrences [5–7].

### The chiropractor in concussion

The chiropractic profession’s contribution to this global, multiple sport and interdisciplinary process warrants scrutiny. As the CISG reviews the latest research and revises its recommendations in preparation for the Sixth International Consensus Meeting on Concussion in Sport (Amsterdam, October 2022), we explore the historical and potential involvement of chiropractors in SRC understanding and management. The primary international sports chiropractic organization composed of national sports councils or national associations, the International Federation of Sports Chiropractic (FICS) report there are 16,085 members with approximately 350 sports chiropractors in Australia, 267 in Canada, 122 in South Africa and roughly 9500 in the United States of America working in collision sports including rugby, hockey, Australian and American football and combat sports [8]. Concussion-related symptoms such as neck pain, headaches and dizziness may respond to chiropractic intervention; therefore, chiropractors should have a clear understanding of how to identify and manage concussion [9]. This is critical to the ‘Recognise, Remove and Refer” mantra often quoted in SRC education.

### Current chiropractic contributions

A PubMed and Google scholar literature review using search terms including “chiropractic/chiropractors” and “sports related concussion” or “concussion”, “chiropractic treatment” and “concussion” searching for chiropractic-specific contributions in the SRC field, suggests a limited contribution by the chiropractic profession that is underweight in absolute terms compared to other medical and allied fields where physiotherapists are playing a leading role. Chiropractors have contributed predominantly in the broader area of systematic reviews of mild traumatic brain injuries (mTBIs) and concussion, with less of a focus on SRC and athletic populations specifically [10–14]. Papers referring to the involvement of chiropractic specifically in SRC primarily centre around knowledge surveys, case series, non-systematic narrative reviews, with only one consensus statement from 2012 [9, 14–20]. Chiropractors involved in the management of SRC appear to be underrepresented in the CISG and International Consensus Conference on Concussion in comparison to other professions. Additionally, there is a dearth of chiropractic-targeted comprehensive post graduate concussion education and training programs or certification processes. With the most prominent and broadly accessible international postgraduate sports chiropractic program, the FICS International Certification in Sports Chiropractic, allocating only 6.5 h out of 80.5 h to head injuries [21].

With chiropractors currently limited in their research investigating assessment and management of upper cervical spine-related post-concussion symptoms in athletes and important knowledge gaps in the recognition and management of SRC, raising professional awareness and knowledge standards of SRC among chiropractors is critically important [9, 17, 22]. This commentary paper discusses the importance of the cervical spine, as well as the key role chiropractors play in recognition, assessment, treatment, and rehabilitation of SRC.

## Concussion mechanism of injury and pathophysiology

SRC is a traumatic brain injury at the mild to moderate end of the brain injury spectrum [3, 23]. The definition of SRC is necessarily broad: ‘*a direct blow to the head, face, neck or elsewhere on the body with an impulse force transmitted to the head’* to account for the many presentations in altered brain function post SRC [24]. For ethical reasons, real measurements of trauma-associated brain pathophysiological alterations are difficult. It is postulated that these biomechanical forces are delivered to the brain; causing damage to the microfilaments and microtubules of the axon leading to decreased *N*-acetyl aspartate (NAA):creatine and NAA:choline ratios, ionic imbalance and calcium overload altering the cellular environment and subsequent brain function in the acute phase [19]. As a result an increased demand for glucose together with an injury-related decrease in resting cerebral blood flow and oxygenation creates an “energy mismatch” [23, 25].

The understanding of this pathophysiological model is primarily derived from animal studies using linear loading forces which are not necessarily representative of SRC, where linear and rotational forces may create a shearing stress on neurons [26, 27]. Furthermore, many biomechanical studies used to establish the findings on rotational acceleration forces rely on modelling used for pedestrian and seated occupants in the automotive industry which lack validity for sport-related head injuries or involve the use of impact sensors which have been found to have limited reliability [3, 27, 28].

## The chiropractor’s role

Modern day SRC management acknowledges the need for oversight by a healthcare practitioner (usually a medical doctor) with expertise in concussion, working in a multidisciplinary team able to address involved domains on an individualised basis. Chiropractors have an opportunity to meaningfully contribute and participate on the multidisciplinary SRC team (and more broadly in mTBI and concussion). The first steps include education, awareness, participating in the “recognise and remove” process and then active involvement in the various stages of management, especially where there may be cervical spine aspects of SRC. Furthermore, the role of allied health including chiropractors becomes essential as SRC often occurs in the absence of a medical practitioner. Additionally, acquiring competencies in vestibular-ocular assessment and rehabilitation should be within the professional scope of a duly trained and licensed chiropractor.

Assessment and rehabilitation of the cervical spine is a key domain of chiropractors. Of particular benefit are combined interventions such as manipulation, mobilization, proprioceptive neuromuscular facilitation (PNF) stretching, soft tissue therapy, acupuncture along with neuromuscular retraining, proprioceptive, range of motion and strengthening exercises [19, 29–31]. Additionally, cervical spine exercises are an essential part of the rehabilitation process with evidence of joint position errors, range of motion and strength deficits in post concussed athletes. Furthermore, reduced neck strength has been associated with higher head acceleration forces, and strengthening the neck muscles—particularly those controlling rotation and lateral flexion—may reduce head acceleration forces decreasing shearing strains on the midline structure of the brain and neck reducing the risk of concussion [29]. Additionally this may in part help to explain why youth and female athletes experience higher rates of concussion [29].

In addition to treatment of the cervical spine, chiropractors must be competent in vestibular-ocular rehabilitation, as approximately 60% of individuals experience vestibular and ocular impairments post SRC [32]. Moreover, evidence indicates those with vestibular ocular reflex (VOR) or tandem gait abnormalities can experience protracted recovery times compared to those with no abnormalities [32, 33]. Tools such as the vestibular ocular motor screening (VOMS) tool and balance error scoring system (BESS) may be useful at identifying potential areas requiring further in depth assessment; especially when they are used as part of a multimodal assessment approach [34–36]. Vestibular rehabilitation has been suggested to be beneficial in reducing dizziness and improving balance in individuals with persistent concussion symptoms although the randomised trial evidence base is yet very limited [13, 37, 38]. It has been proposed that therapy should focus on habituation, gaze stabilization, head-eye coordination, balance and mobility exercises with graduated challenges to the base of support [13, 39, 40]. Ocular therapy in some cases may require more specialized interventions such as speeded saccadic eye movements, visual pursuit, tracking tasks, reading tasks and visual attention tasks [32, 41].

Further development of the cervical spine injury assessment protocol for use in either the SCAT or in-office assessment protocols is another area in which chiropractors are well placed to contribute [42]. Chiropractors have been at the forefront of developing whiplash grading systems to establish clinical outcomes; in addition to further improving our understanding of the course and prognostic factors associated with neck pain and whiplash [43, 44]. Together with knowledge and experience in assessing and managing cervicogenic complaints, the profession is well positioned to assist in establishing guidelines for best practice management of neck pain [42]. Indeed, chiropractic clinicians and researchers working intensely in the area of SRC may consider contributing to and collaborating in the CISG to provide input into the assessment and management of the cervical spine in SRC from a chiropractic perspective.

## The role of the cervical spine in concussion

The kinematics of a head impact in sport involves rapid displacement and rotation of the head; with concurrent neck tension, shearing and bending especially in rotation and lateral flexion, giving rise to the potential for injury of the cervical spine [45, 46]. At present it is thought the shearing and straining forces during a head impact may affect the deep midline structures of the brain such as the midbrain, fornix and the corpus callosum correlating with loss of consciousness, cognitive related and memory dysfunction often seen in concussed athletes [47, 48]. However, those symptoms commonly associated with SRC including headache, dizziness, nausea, neck pain, fatigue, irritability and blurred vision do not correlate with strain of the deep midline structures and may in part be attributed to the upper cervical spine [46, 49]. Symptoms occurring after a head impact, often attributed to mTBI, may also be caused by cervical spine/vestibular injury [49].

Although research in this space is still very limited and needs to be better designed and conducted, it is hypothesized that the upper cervical spine may be more likely to contribute to symptoms than the lower cervical spine; C1–C3 provide a greater contribution to sensorimotor control [46, 50]. Additionally, the deep suboccipital muscles have abundant cervical afferents and more slow twitch fibres making them well suited as proprioception monitors [50, 51]. Furthermore, reflex connections between the neck, visual and vestibular systems relating to head and eye movement control, as well as postural control arise from the upper cervical spine [50]. More robust evidence is needed to substantiate theories that concussion-related trauma may lead to abnormal somatosensory afferents arising from the muscle spindles, joints, pain receptors or nerve roots of the cervical spine contributing to symptoms like headache, dizziness and vertigo [51, 52]. It has been suggested that aberrant cervical somatosensory information may directly affect the cervical reflexes affecting the vestibular reflex and ocular responses; where irregular cervical information is mismatched with normal vestibular and visual input subsequently resulting in dizziness, disorientation, and balance disturbances. This lends further support to the notion that not all manifestations of SRC are necessarily from a brain injury [51, 52].

### Assessment of concussion-like symptoms

Athletes who exhibit symptoms such as neck pain, headaches, dizziness, blurred vision, nausea following a collision impact are automatically diagnosed with a SRC; however, these symptom manifestations closely overlap with a mechanical injury to the cervical spine [49, 51–53]. Furthermore, cervical proprioception plays an important role in neck injury and disequilibrium [54]. A thorough assessment of the cervical spine is an important aspect of SRC assessment. Traditionally, this involves a battery of tests including range of motion, strength (flexion, extension, lateral flexion and rotation) and motor control, postural control, gaze stability and balance, both static and dynamic balance, functional gait and benign paroxysmal positional vertigo [29, 51, 53, 55]; however, little is empirically known about the diagnostic accuracy of these tests. Table 1 provides a list of cervical spine assessment tests and a summary of their psychometric properties evidence base.

**Table 1:** Cervical spine assessment tests and evidence of their psychometric properties

Test and study	Population (study size)	Intra-rater reliability (ICC)	Inter-rater reliability (ICC)	Validity		
				Sensitivity (%)	Specificity (%)	PPV (%)
*Joint position error*						
Alahmari [56]	69	.62–.84	.74.78	NR	NR	NR
*Smooth pursuit neck torsion*						
Majcen [57]	64	.497–.751	NR	NR	NR	NR
Daly [58]	40	NR	NR	27.3	79.3	NR
*Cervical flexion endurance*						
Juul [59]	63	.68–.75	.70–.73	NR	NR	NR
Selistre [60]	219/193	.85	.75	NR	NR	NR
*Anterolateral cervical muscle strength*						
Selistre [60]	200	.49	NR	NR	NR	NR
*Head thrust/impulse*						
Singh [61]	40	≥ .76	NR	NR	NR	NR
Jacobson [62]	116	NR	NR	27	85	NR
*Cervical flexion rotation*						
Hall [63]	40	NR	.93	90	88	NR

*NR* Not reported

The cervical spine assessment in the current SCAT 5 is likely to be insufficient as it only assesses for pain and active range of motion [64]. A more rigorous cervical spine assessment protocol is recommended; as post-concussion signs related to the cervical spine include increased neck pain, headaches and dizziness in those with pre-existing symptoms; as well as new onset of neck pain, headaches and dizziness; reduced range of motion in at least one direction, reduced anterolateral neck strength, reduced neck endurance, impaired dual task gait and dynamic stability and impaired neck position sensing [51–53, 55].

Consideration must also be given to the sensorimotor system which includes the vestibular, ocular, and proprioceptive systems which can undergo disruption during traumatic impacts associated with SRC. Evidence suggests that mechanisms involving low speed forces, with no head impact, concussion or loss of consciousness, are more likely to produce symptoms due to trauma of the cervical spine joints and muscle related receptors rather than the central nervous or peripheral vestibular systems [41]. Furthermore, dizziness which maybe a symptom of disruption to the vestibular and/or ocular motor systems is reported by 50% of concussed athletes and is associated with a 6.4 times increased risk of a protracted recovery; 40% of athletes subjectively report balance disturbances in the first week of a SRC; 30% report visual disturbances during the first week [65]. Although VOMS screening tools are not a standalone diagnostic tool, they demonstrate internal consistency and sensitivity at diagnosing those with concussion from healthy individuals; with elements of the VOMS significantly related to protracted recovery times [34, 65]. Assessment of all domains potentially contributing to the spectrum of post-injury symptoms is pertinent and familiarisation with ocular and vestibular assessment is crucial; Table 2 provides the key ocular and vestibular tests and evidence of their psychometric properties.

**Table 2:** Ocular and vestibular assessment tests and evidence of their psychometric properties

Test and study	Population (study size)	Intra-class reliability (ICC)	Validity		
			Sensitivity (%)	Specificity (%)	PPV (%)
*Vertical and horizontal saccades*					
Hunfalvay [66]	195	NR	77 (horizontal) 64 (vertical)	78 (horizontal) 65 (vertical)	NR
*Dynamic visual acuity*					
Kaufman [67]	50	.77 (yaw) .725 (pitch)	NR	NR	NR
*Convergence*					
Yorke [35]	105	.95	NR	NR	NR
Pearce [68]	78	.95–.98	NR	NR	NR
*Hallpike*					
Halker [69]	61	NR	79	75	95.8
*BESS*					
Murray [70]		.87	34	91–96	NR
Oldham [71]	76	NR	44.7	50	NR
*Modified BESS*					
Oldham [71]	76	NR	47.4	63.2	NR
*Tandem gait*					
Oldham [71]	76	NR	63.2	60.5	NR
*Dual-task tandem gait*					
Howell [72]	32	.84	NR	NR	NR
Van Deventer [73]	170	NR	22.6	95.1	75

## Conclusion

SRC is prevalent and widespread occurring across a variety of sports. Chiropractors often work with teams involved in contact and collision sports and it is reasonable to expect that patients suffering from post-concussion symptoms including headache, dizziness, and neck pain may present to a chiropractor in the subsequent days and weeks following a SRC injury. Furthermore, chiropractors work with many sports teams making it prudent that they understand the modern, evidence-based, domain-orientated approach to concussion assessment and management. This context presents an opportunity for chiropractic clinicians and scientists to contribute and become involved in the SRC field more meaningfully. To do so and considering the knowledge gaps in the assessment and management of SRC, we recommend that formal and comprehensive concussion training based on evidence and best practice be included at both under- and postgraduate chiropractic training levels. In addition, chiropractors can contribute significantly, in the assessment and management of cervicogenic pain in the concussed athlete population. Finally, the profession should seek to collaborate on the advancement of the current SCAT5 cervical spine evaluation guidelines.

## Data Availability

Not applicable.

## Abbreviations

CISG: Concussion in Sport Group
SRC: Sports related concussion
SCAT: Sports concussion assessment tool
VOR: Vestibular ocular reflex
VOM: Vestibular ocular motor screening
BESS: Balance error scoring system
BBPV: Benign paroxysmal positional vertigo
